# Glial Cells as Emerging Therapeutic Targets in Neurodegenerative Diseases: Mechanistic Insights and Translational Perspectives

**DOI:** 10.3390/cells14191497

**Published:** 2025-09-24

**Authors:** Thirupathirao Vishnumukkala, Che Mohd Nasril Che Mohd Nassir, Zaw Myo Hein, Prarthana Kalerammana Gopalakrishna, Barani Karikalan, Aisyah Alkatiri, Saravanan Jagadeesan, Venkatesh R. Naik, Warren Thomas, Mohamad Aris Mohd Moklas, Mohd Amir Kamaruzzaman

**Affiliations:** 1 Department of Human Anatomy, Faculty of Medicine and Health Sciences, Universiti Putra Malaysia, Serdang 43400, Selangor, Malaysia; thirupathirao@imu.edu.my (T.V.); aris@upm.edu.my (M.A.M.M.); 2 Human Biology Department, School of Medicine, IMU University, Bukit Jalil, Kuala Lumpur 57000, Malaysia; prarthana@imu.edu.my (P.K.G.); 00000038358@student.imu.edu.my (A.A.); 3 Department of Anatomy and Physiology, School of Basic Medical Sciences, Faculty of Medicine, Universiti Sultan Zainal Abidin, Kuala Terengganu 20400, Terengganu, Malaysia; nasrilnassir@unisza.edu.my; 4 Department of Basic Medical Sciences, College of Medicine, Ajman University, Ajman P.O. Box 346, United Arab Emirates; z.hein@ajman.ac.ae; 5 Center of Medical and Bio-Allied Health Sciences Research (CMBHSR), Ajman University, Ajman, United Arab Emirates; 6 Department of Pathology, Faculty of Medicine, MAHSA University, Bandar Saujana Putra, Jenjarom 42610, Selangor, Malaysia; barani@mahsa.edu.my; 7 Department of Anatomy, School of Medicine, Taylor’s University, Lakeside Campus, Subang Jaya 47500, Selangor, Malaysia; saravanan.jagadeesan@taylors.edu.my; 8 Department of Preclinical Sciences, Faculty of Medicine and Health Sciences, University Tunku Abdul Rahman, Jalan Sungai Long, Bandar Sungai Long, Kajang 31900, Selangor, Malaysia; venkatesh@utar.edu.my; 9 School of Medicine, Royal College of Surgeons in Ireland—Medical University of Bahrain, Muharraq Governate, Kingdom of Bahrain; wathomas@rcsi-mub.com; 10 Department of Anatomy, Faculty of Medicine, Universiti Kebangsaan Malaysia, Jalan Yaacob Latif, Bandar Tun Razak, Cheras, Kuala Lumpur 56000, Malaysia

**Keywords:** glial cells, neurodegenerative disease, neuroinflammation, neurodegeneration, therapy

## Abstract

Neurodegenerative diseases such as Alzheimer’s disease (AD), Parkinson’s disease (PD), Huntington’s disease, multiple sclerosis, and amyotrophic lateral sclerosis share converging mechanisms of neuronal dysfunction, including protein aggregation, oxidative stress, and chronic neuroinflammation. Glial cells, once considered passive supporters, are now recognized as central drivers of these processes, offering both pathogenic triggers and therapeutic opportunities. Yet, despite compelling preclinical evidence, the translation of glial-targeted therapies into clinical success has been limited. This review provides a critical synthesis of current knowledge by examining therapeutic strategies through the lens of their translational challenges and failures. This narrative review highlights how interspecies variability of glial phenotypes, shifting neuroprotective versus neurotoxic states, limited biomarker stratification, and delivery barriers have constrained trials, such as anti-triggering receptor expressed on myeloid cells 2 (anti-TREM2) antibodies in AD and glial cell line-derived neurotrophic factor (GDNF) in PD. By analyzing these obstacles across major neurodegenerative disorders, this review argue that the next stage of glial medicine requires precision approaches that integrate stage-specific phenotyping, biomarker-guided patient selection, and innovative delivery platforms. Understanding not only what has been tried but why translation has stalled is essential to chart a roadmap for effective, disease-modifying glial therapies in the aging brain.

## 1. Introduction

Neurodegeneration refers to the progressive structural and functional loss of neurons, leading to a deterioration of cognitive, motor abilities, and overall quality of life. It is the defining hallmark of major neurodegenerative diseases, such as Alzheimer’s disease (AD), Parkinson’s disease (PD), amyotrophic lateral sclerosis (ALS) and multiple sclerosis (MS) [1]. The underlying pathophysiology is complex and multifactorial, involving processes such as oxidative stress, mitochondrial dysfunction, protein misfolding, excitotoxicity, and chronic neuroinflammation [2]. For instance, α-synuclein aggregation in PD fosters Lewy body formation and neuronal loss [3], while in AD, amyloid-β deposition and tau pathology interact with glial dysfunction to accelerate synaptic failure. Neuroinflammation, particularly driven by chronically activated microglia and reactive astrocytes, exemplifies the double-edged nature of glial responses: acutely beneficial for repair, but detrimental when persistent [4].

The established epidemiology of these neurodegenerative diseases reveals a growing public health concern, particularly with our aging populations. The prevalence of these disorders is increasing, with estimates suggesting that by 2050, the number of individuals with dementia alone could reach 152 million globally [5]. Importantly, this rising prevalence cannot be explained by age alone; it reflects the interaction of genetic predispositions, environmental exposures, and modifiable lifestyle factors such as sleep quality, diet, and vascular health [6,7,8]. For example, disrupted sleep has been associated with impaired clearance of misfolded proteins via the glymphatic system, thereby heightening neurodegenerative risk [7]. Similarly, vascular comorbidities, including hypertension and diabetes, exacerbate neuronal vulnerability, illustrating the interconnectedness of systemic health and neurological decline [8]. These trends highlight the urgency of identifying disease-modifying strategies that extend beyond symptomatic management.

In this context, glial cells have emerged from the background of neuroscience to become central players in both the initiation and progression of neurodegeneration. Astrocytes, microglia, and oligodendrocytes not only maintain homeostasis, synaptic function, and metabolic support under physiological conditions but also act as active mediators of neuronal injury when their functions are dysregulated. Astrocytic failure in glutamate clearance promotes excitotoxicity; microglial hyperactivation sustains chronic inflammation; and oligodendrocyte loss undermines axonal Integrity [4]. Aging further compounds these alterations by biasing glia toward pro-inflammatory, less reparative states, thereby magnifying disease susceptibility [7]. These insights have shifted the therapeutic landscape, positioning glia not as passive bystanders but as attractive targets for intervention across multiple disorders.

However, despite strong mechanistic rationale and encouraging preclinical findings, the clinical translation of glial-targeted therapies has been fraught with challenges. Trials of anti-triggering receptor expressed on myeloid cells 2 (anti-TREM2) antibodies in AD [9], glial cell line-derived neurotrophic factor (GDNF) in PD [10], and excitatory amino acid transporter 2 (EAAT2)-enhancing strategies in amyotrophic lateral sclerosis (ALS) [11] have yielded mixed or disappointing outcomes, reflecting broader obstacles in the field. Key barriers include interspecies variability of glial gene expression, the dynamic and disease-stage-dependent nature of glial phenotypes, the absence of reliable biomarkers for patient stratification, and difficulties in achieving targeted delivery across the blood–brain barrier (BBB). These failures are not setbacks alone but valuable lessons, highlighting the need for precision approaches that align therapeutic strategies with the context-specific roles of glia.

This review, therefore, seeks to critically evaluate glial cells as therapeutic targets across major neurodegenerative disorders. Unlike descriptive overviews, we aim to synthesize insights from both successful and failed clinical and preclinical attempts, drawing attention to the translational bottlenecks that have limited progress. By applying this lens to AD, PD, HD, MS, and ALS, we highlight converging mechanisms of glial dysfunction, contextualize why interventions have struggled to move beyond the laboratory, and propose how biomarker-guided, stage-specific, and delivery-focused approaches may advance the next generation of glial-based therapies. In doing so, we argue that understanding why translation has stalled is as critical as identifying new targets, and that overcoming these limitations is essential to achieving disease-modifying interventions for currently intractable neurodegenerative diseases.

## 2. Molecular Aspect of Glial Cells in Neurodegeneration

Glial cells, often overlooked in the shadow of their more renowned neuronal counterparts, play a crucial part in the proper functioning of the central nervous system (CNS). Contrary to the prevailing belief that they merely provide structural and supportive roles, recent studies have revealed the diverse and indispensable functions of glial cells in various phases of nervous system development and function [12]. Glial cells, which greatly outnumber neurons in the human brain, originate from a common progenitor cell during embryonic development [12]. The three main types of glial cells in the CNS are astrocytes, oligodendrocytes, and microglial cells.

Astrocytes maintain the extracellular environment, regulate neurotransmitter levels, and contribute to the integrity of the BBB [13]. Oligodendrocytes are responsible for myelinating CNS axons, ensuring rapid signal transmission and metabolic support [14]. Microglia, the resident immune cells of the CNS, continuously survey the neural microenvironment, responding to injury and infection through phagocytosis, antigen presentation, and cytokine release [15]. Beyond these foundational roles, recent discoveries have revealed that glial cells actively participate in complex neural processes, including synaptic modulation, neurovascular coupling, and plasticity. Astrocytes can influence neuronal excitability via glutamate uptake and calcium signaling, while microglia shape synaptic architecture through pruning during development and disease [16]. These dynamic functions underscore glia not as passive supporters, but as vital regulators of cognition, learning, and memory. In neurodegenerative diseases, these functional roles become pathologically altered.

Across AD, PD, HD, MS, and ALS, glial cells contribute to neuronal degeneration through several converging mechanisms: (i) chronic neuroinflammation, whereby prolonged activation of microglia and reactive astrocytes leads to the release of pro-inflammatory cytokines, including tumor necrosis factor alpha (TNF-α), and interlkeukins (IL-1β, and IL-6), fostering a neurotoxic environment that impairs synaptic function and accelerates neuronal death [17]; (ii) oxidative stress: activated glia generates reactive oxygen species (ROS), exacerbating mitochondrial dysfunction in both neurons and glial cells, and promoting cellular damage [18]; (iii) impaired proteostasis: Defective autophagy and lysosomal pathways hinder the clearance of misfolded proteins such as amyloid-β (Aβ), α-synuclein, and mutant huntingtin, promoting toxic aggregation and spreading pathology [19]; (iv) metabolic dysfunction: Mitochondrial impairments in astrocytes and oligodendrocytes reduce their capacity to meet the high energy demands of neurons, especially under conditions of stress or injury [20]; and (v) excitotoxicity and synaptic dysregulation: Reduced expression of glutamate transporters, such as EAAT2 in astrocytes, leads to elevated extracellular glutamate levels and excitotoxic neuronal injury, disrupting communication and plasticity [21].

Collectively, these glial-mediated mechanisms form a pathological feedback loop that amplifies neurodegeneration. Notably, many of these dysfunctions are exacerbated with aging, which alters glial phenotypes toward pro-inflammatory and less reparative states. Recognizing the shared molecular pathways of glial dysfunction across neurodegenerative disorders provides a framework for identifying therapeutic targets that are broadly applicable and potentially disease-modifying.

## 3. Role of Glial Cells in Neurodegeneration

Glial cells play a dual and dynamic role in the pathogenesis of neurodegenerative diseases. While essential for maintaining CNS homeostasis, metabolic support, and synaptic function, they can also become key contributors to neuronal dysfunction and degeneration under pathological conditions [17,22]. The phenotypic shift from neuroprotective to neurotoxic states, commonly triggered by aging, oxidative stress, or protein aggregates, underpins much of the chronic inflammation and tissue damage observed in AD, PD, MS, HD, and ALS. Moreover, astrocytes are known for their supportive roles in the CNS, by providing both metabolic and trophic support to the neurons. They protect neurons from cytotoxicity, as demonstrated in studies where glial U87 cells mitigated the detrimental effects of radiation on neuronal SH-SY5Y cells by reducing oxidative stress and apoptosis [23]. Furthermore, astrocytes can modulate neuroinflammation, which is a significant aspect of neurodegenerative diseases. For instance, when astrocytes are activated, this leads to the secretion of various inflammatory cytokines, which can exacerbate neuronal damage in AD and PD [24]. From the perspective of AD, astrocytes and microglia are implicated in the clearance of amyloid-beta (Aβ) peptides, although defective glial function also contributes to disease progression [25].

On the other hand, microglia, which form the resident immune cells of the CNS, play a pivotal part in neuroinflammatory responses. They can become hyperactivated in neurodegenerative conditions, leading to increased secretion of pro-inflammatory cytokines, which can further drive neurodegeneration [26]. For instance, in models of tauopathy, microglia have been shown to drive neurodegeneration through their interactions with apolipoprotein E (ApoE), highlighting the complex interplay between glial cells and neuronal cell health [27]. This is further highlighted by the dysfunction of microglia, referred to as “gliopathy,” being associated with the neuronal dysfunction found in chronic pain and neuroinflammation [28].

Another important glial cell is the oligodendrocytes, which is responsible for myelinating axons, and contributes to neuronal survival and function. Their dysfunction can lead to demyelination and subsequent neuronal degeneration, as seen in diseases like multiple sclerosis. Studies have shown that glial progenitor cells can be affected by viral infections, which may interfere with their differentiation and contribute to neurodegenerative processes [29]. Moreover, the interaction between glial cells and neurons is crucial for maintaining synaptic integrity and function. Disruption of these interactions can lead to neurodegenerative changes, as observed in models of nerve damage where glial cells fail to support neuronal health adequately [30].

Altogether, glial cells are integral to both the maintenance of neuronal health and the progression of neurodegenerative diseases. Their normal protective roles can be compromised in pathological conditions, leading to a cycle of inflammation and neuronal damage. A clear understanding is essential of the dual role of glial cells in neurodegeneration, for the development of therapeutic strategies aimed at mitigating their unfavorable effects while boosting their protective functions. Table 1 summarizes the common glial-mediated mechanisms implicated across major neurodegenerative diseases, illustrating how diverse clinical phenotypes converge on shared pathological processes.

## 4. Alzheimer’s Disease (AD)

AD is a disorder characterized by progressive neurodegeneration and is the most common cause of dementia worldwide, accounting for 60–70% of dementia cases [75]. In AD, there is a gradual decline in cognitive functions, including memory, reasoning, and executive skills, ultimately impairing the individual’s daily activities [76]. AD predominantly affects older adults, with age being the primary risk factor, though genetic, environmental, and lifestyle factors also contribute to the onset and progression of the disease [77].

Pathologically, AD is defined by the accumulation of Aβ plaques and neurofibrillary tangles composed of hyperphosphorylated tau protein in the brain [78]. These features are the hallmarks of AD and are associated with widespread loss of neurons, synaptic dysfunction, and chronic neuroinflammation [79]. In addition, vascular changes, impaired mitochondrial function, and oxidative stress have been implicated in the disease’s pathology, further complicating our understanding of the underlying mechanisms contributing to AD pathology [80]. Despite the substantial advances that have been made in understanding the various molecular and cellular processes involved in AD, accurate etiology remains elusive, and there is currently no cure. Existing pharmaceutical drugs, such as cholinesterase inhibitors and N-methyl-D-aspartate (NMDA) receptor antagonists, primarily offer symptomatic relief rather than aiming to treat the root causes of the disease [81]. Consequently, AD remains a significant public health challenge, with substantial socio-economic and emotional burdens on individuals, caregivers, and healthcare systems.

Recent research has stressed the critical role of glial cells, including astrocytes, microglia, and oligodendrocytes, in the disease process of AD [82]. These cells, once considered mere support systems for neurons, are now recognized as active contributors to neurodegeneration and potential therapeutic targets.

### 4.1. Glial Dysfunction and Pathogenesis

The classical features of AD are the progressive neurodegenerative changes, cognitive decline, loss of memory, and changes in behavior.

The extracellular accumulation of Aβ peptides results from the proteolytic cleavage of amyloid precursor protein (APP) by the β-secretase and γ-secretase enzymes [78]. Misfolded Aβ aggregates form oligomers, fibrils, and eventually insoluble plaques, disrupting neuronal function and triggering downstream cytotoxic cascades. Aβ oligomers are particularly neurotoxic, impairing synaptic transmission and inducing oxidative stress [83,84].

Another defining feature is the hyperphosphorylated tau protein, which aggregates intracellularly, forming neurofibrillary tangles (NFTs). Tau, which is a microtubule-associated protein, becomes dysfunctional when hyperphosphorylated, thus giving rise to microtubule destabilization, axonal transport disruption, and neuronal apoptosis [85]. NFTs correlate strongly with disease progression and severity [86].

Chronic activation of microglial cells and astrocytes plays a central role in AD-associated neuroinflammation. Though initially protective, the prolonged neuroinflammation exacerbates neuronal damage through over-production of pro-inflammatory cytokines and ROS and complement system activation [79]. Microglia also fail to effectively clear Aβ deposits, contributing to the persistence of plaques. In addition, the neuronal synaptic plasticity is impaired by the Aβ oligomers, which disrupt long-term potentiation (LTP), while it enhances long-term depression (LTD), which are crucial mechanisms for learning and memory establishment. Dysregulation of glutamate signaling due to Aβ toxicity leads to excitotoxicity, causing neuronal injury and death [87].

Mitochondrial dysfunction and oxidative stress are also prominent in AD. Mitochondria in affected neurons exhibit structural and functional abnormalities, leading to impaired energy metabolism. Excessive ROS production damages lipids, proteins, and DNA, creating a vicious cycle involving oxidative stress and further mitochondrial damage [43]. Cerebrovascular dysfunction associated with AD, which includes a reduction in cerebral blood flow and disruption of the BBB, further aggravates the neuronal damage and impairs Aβ clearance. Cerebral amyloid angiopathy (CAA), which is characterized by Aβ deposition in the walls of cerebral blood vessels, compromises vascular integrity and function [88].

Loss of neurotransmitter systems, particularly the degeneration of basal forebrain cholinergic neurons, contributes to cognitive and memory deficits. While reduced acetylcholine levels, along with disruptions in glutamatergic and serotonergic pathways, further impair neuronal communication [89]. Genetic factors have an important role in AD. Mutations that occur in APP, PSEN1, and PSEN2 are associated with familial forms of the disease, leading to enhanced production of Aβ42, a highly aggregation-prone isoform of Aβ [90]. The presence of the ApoE ε4 allele significantly raises the risk of sporadic AD, influencing Aβ metabolism and clearance [91].

Overall, the multifactorial nature of pathophysiology involved in AD underscores the complexity of the disorder and highlights potential therapeutic targets, including Aβ and tau reduction, modulation of neuroinflammation, and neuroprotection.

### 4.2. Glial-Targeted Interventions and Imaging Biomarkers

Glial cells are now recognized as central mediators of AD pathology. Microglia and astrocytes respond dynamically to Aβ accumulation and tau pathology, exhibiting both protective and neurotoxic behaviors depending on disease stage and cellular context.

Microglia contribute to Aβ clearance through phagocytosis but also propagate inflammation via the release of cytokines and ROS upon chronic activation [56]. Their function is modulated by innate immune receptors, such as toll-like receptor 4 (TLR4) and NLR family pyrin domain containing 3 (NLRP3). TLR4 activation triggers Nuclear factor kappa-light-chain-enhancer of activated B cells (NF-κB) signaling and cytokine production, while NLRP3 inflammasome activation enhances caspase-1-mediated IL-1β release, promoting neuronal injury [31]. Inhibition of NLRP3 reduces Aβ deposition and cognitive deficits in transgenic models.

The TREM2-DAP12 signaling pathway is crucial for microglial transition to a disease-associated microglia (DAM) phenotype that enhances Aβ phagocytosis. Loss-of-function mutations in TREM2 impair this protective mechanism and increase AD susceptibility. Agonistic anti-TREM2 antibodies (e.g., AL002) are currently in clinical trials aiming to augment microglial resilience [9].

Astrocytes, in response to Aβ and neuronal stress, become reactive, upregulating GFAP and pro-inflammatory mediators. While moderate reactivity may support tissue repair, excessive activation perpetuates inflammation and impairs neuronal function. Astrocytic dysfunction also contributes to glutamate excitotoxicity via downregulation of EAAT2, and loss of brain-Derived Neurotrophic Factor (BDNF) through PI3K/Akt pathway suppression further undermines synaptic integrity and BBB function [32,92].

**Figure 1:**
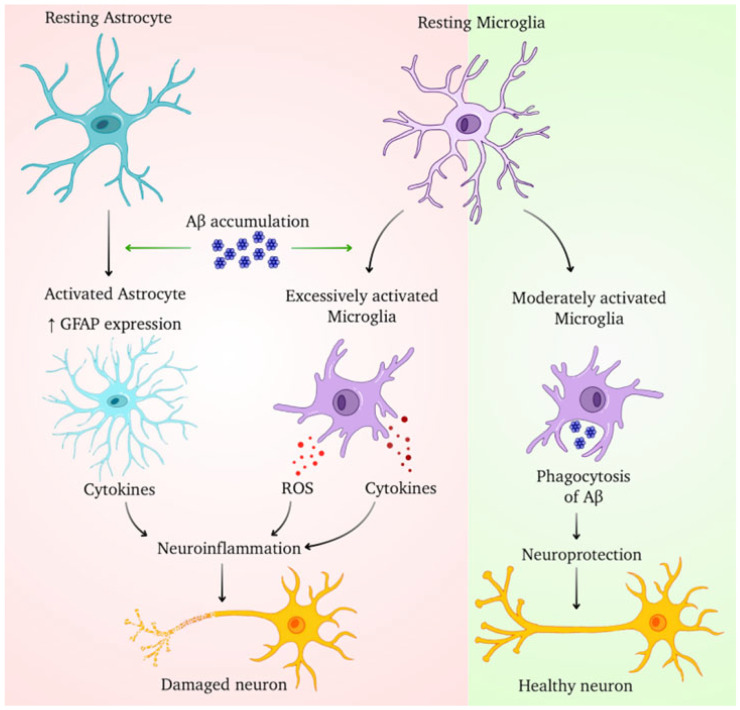
The picture shows how glial cells respond differently to amyloid-β (Aβ) buildup in the brain. On the left, Aβ buildup activates astrocytes (as seen by increased GFAP expression), leading to the release of pro-inflammatory cytokines. Concurrently, excessive microglial activation causes the formation of reactive oxygen species (ROS) and cytokines, which leads to neuroinflammation and neuronal injury. In contrast, the right panel demonstrates a protective mechanism where moderately active microglia efficiently phagocytose Aβ aggregates, providing neuroprotection and maintaining neuronal integrity.

In vivo imaging using translocator protein (TSPO) PET tracers (e.g., [^11^C]PK11195, [^18^F] DPA-714) reveals elevated glial activation in early and prodromal AD, correlating with amyloid burden and cognitive decline [93]. Longitudinal PET studies show that persistent microglial activation is associated with hippocampal atrophy and accelerated cognitive deterioration, reinforcing the link between glial activity and disease progression. Figure 1 illustrates microglial and astrocytic contributions to Aβ pathology, emphasizing their dual roles in clearance and inflammation [94].

Glial-targeted therapies are under investigation, including CSF1R inhibitors to limit microglial proliferation, p38 MAPK and NF-κB inhibitors to suppress astrocytic inflammation, and cell-based approaches to restore glial function. Transplantation of glial progenitor cells in animal models has shown promise in reducing Aβ burden and improving cognition [95].

In sum, glial cells in AD act as both regulators and effectors of disease pathology. Their dual roles, neuroprotective in early stages and neurotoxic upon chronic activation, make them compelling therapeutic targets. A deeper understanding of glial phenotypes, signaling pathways, and their interaction with neuronal and vascular systems may yield novel strategies for disease modification in AD.

Despite the compelling rationale for targeting microglia and astrocytes in AD, translation into effective therapies has been challenging. Trials of TREM2 agonistic antibodies, for example, underscore the difficulty of moving from preclinical promise to clinical efficacy. Interspecies variability in microglial gene expression raises questions about how faithfully murine models reflect human biology, while the absence of robust biomarkers to stratify patients according to microglial activation state or TREM2 genotype dilutes potential treatment effects. Similarly, astrocytic modulators face the challenge of balancing protective versus toxic reactivity, a dynamic that changes across disease stages (REF). These translational barriers reflect the broader challenges of glial-targeted medicine and highlight the urgent need for precision approaches, where patient selection, biomarker monitoring, and longitudinal phenotyping guide the use of glial-targeted interventions in AD. These observations reflect broader translational barriers discussed in Section 9.1, which detail cross-species differences, phenotypic plasticity, biomarker limitations, and delivery challenges.

## 5. Parkinson’s Disease (PD)

PD is a disorder that affects motor function; it is chronic, progressively neurodegenerative, and is ranked in frequency next to AD in the list of neurodegenerative diseases, with an estimated prevalence of 1% in individuals over the age of 60 years [96]. The clinical presentation of PD shows cardinal motor symptoms, which include bradykinesia, resting tremors, rigidity, and postural instability, and a wide range of non-motor symptoms, such as cognitive impairment, depression, and autonomic dysfunction [97]. These symptoms profoundly impact patients’ quality of life and increase caregiver burden.

The key pathological marker of PD is the selective degeneration of dopaminergic neurons present in the substantia nigra pars compacta (SNpc), which results in dopamine depletion in the striatum [98]. Additionally, the observation of Lewy bodies, which are intracellular aggregates of misfolded α-synuclein, is a defining feature of the disease [99]. Though the exact etiology of PD remains uncertain, it is believed to be the result of a complex interaction between genetic susceptibility and environmental factors that include pesticide exposure, head trauma, and aging [100].

Currently, PD has no cure, and available pharmaceutical interventions like levodopa and other dopamine agonists primarily address the symptoms without altering disease progression [101]. The search for disease-modifying treatments has led to an increased focus on understanding the impact of neuroinflammation, mitochondrial dysfunction, and oxidative stress in PD pathogenesis [102].

Recent research has also underlined the role of glial cells, with particular focus on astrocytes and microglia in PD progression [33]. These cells are recognized as active contributors to neurodegeneration through various mechanisms, such as the liberation of inflammatory cytokines and impaired phagocytosis of α-synuclein aggregates. Targeting glial cell dysfunction represents an encouraging avenue for therapeutic intervention and offers hope to alter the trajectory of this debilitating disease.

### 5.1. Pathophysiology of PD

PD is a progressive degenerative disorder, primarily affecting the dopaminergic neurons in the SNpc, which is a critical area in the brain involved in movement regulation through the modulation of different basal ganglia circuits. The damage and the subsequent dopamine depletion in the striatum, impair the balance between the direct and indirect pathways involving the basal ganglia. This results in the motor symptoms observed in PD, including bradykinesia, rigidity, resting tremors, and postural instability [102]. The loss of dopamine in PD results in a relative dominance of the indirect pathway, which inhibits movement, and an impaired functioning of the direct pathway, which facilitates movement. This imbalance leads to the motor symptoms seen in PD [103].

To add to the existing dopaminergic degeneration, the accumulation of α-synuclein, a protein involved in synaptic vesicle function, is a critical pathological feature of PD. Misfolded α-synuclein aggregates form Lewy bodies, which are intracellular inclusions that disrupt cellular function, contribute to neurotoxicity, and promote cell death. Lewy bodies, which are usually seen in the substantia nigra, can also be observed in other parts of the brain as the disease progresses, contributing to the non-motor symptoms of PD, including cognitive impairment and autonomic dysfunction [99].

Neuroinflammation is another prominent aspect of PD pathophysiology. Microglia are activated in response to neurodegeneration. Prolonged microglial activation contributes to neuroinflammation, thus exacerbating neuronal injury and accelerating disease progression [33]. Astrocytes may also contribute to neuroinflammation in PD, affecting the Blood–Brain Barrier, glutamate regulation, and neuronal survival [48].

Dysfunction of neuronal mitochondria and oxidative stress are also implicated in the pathophysiology of PD. In PD, mitochondrial dysfunction leads to a reduction in ATP production and an increase in ROS, with subsequent damage to cellular components and structures, including membranes. This oxidative damage brings about the degeneration of dopaminergic neurons in the substantia nigra [44].

Genetic factors have also been implicated in the pathophysiology of PD, with several genes, such as LRRK2, PARK7, and SNCA (which encodes α-synuclein), being linked to familial forms of PD. These genetic mutations result in defective protein degradation pathways, mitochondrial function, and disruption of cellular homeostasis, further contributing to neuronal degeneration [100]. As PD progresses, neurodegeneration spreads to other regions of the brain, such as the cerebral cortex, limbic system, and brainstem, contributing to cognitive and behavioral changes, sleep disturbances, and autonomic dysfunction. This widespread neurodegeneration in the brain accounts for the complex array of symptoms observed during advanced stages of the disease, beyond the motor impairments [104].

### 5.2. Glial Mechanisms in PD Pathogenesis

The importance of glial cells in the pathogenesis of PD and the potential for their inclusion in future treatment regimens has gathered significant attention in recent years, all the while research continues to elucidate the complex interactions between glial cells and neurons in the pathophysiology of this neurodegenerative disorder. This shift in understanding has opened new avenues for therapeutic interventions aimed at modulating glial cell functions to ameliorate the symptoms and progression of PD. Glia-induced neuroinflammation worsens the dopaminergic neuron degeneration in the substantia nigra [105,106], especially if this becomes chronic [107].

GDNF is a key player in the neuroprotective actions of glial cells. Originally identified for its ability to support dopaminergic neurons, GDNF also exerts protective effects against neurotoxic insults in various models of PD [10]. Clinical trials have demonstrated that GDNF administration can bring about significant improvements in motor function in patients with PD, underscoring its potential as a therapeutic agent [108]. However, the efficacy of GDNF-based therapies may be limited by the challenges associated with their delivery and the need for sustained release mechanisms, such as microspheres that can provide long-term neuroprotection [109].

Moreover, the interaction between glial cells and α-synuclein is critical for understanding the disease’s pathology. Recent studies have shown that astrocytes can uptake and clear α-synuclein aggregates from the extracellular space, suggesting a protective role in maintaining neuronal health [57]. However, prolonged exposure to α-synuclein can lead to astrocytic dysfunction, contributing to neuroinflammation and neuronal death [58]. This indicates that therapeutic strategies aimed at enhancing the clearance capabilities of astrocytes or modulating their response to α-synuclein exposure could hold promise for PD treatment.

**Figure 2:**
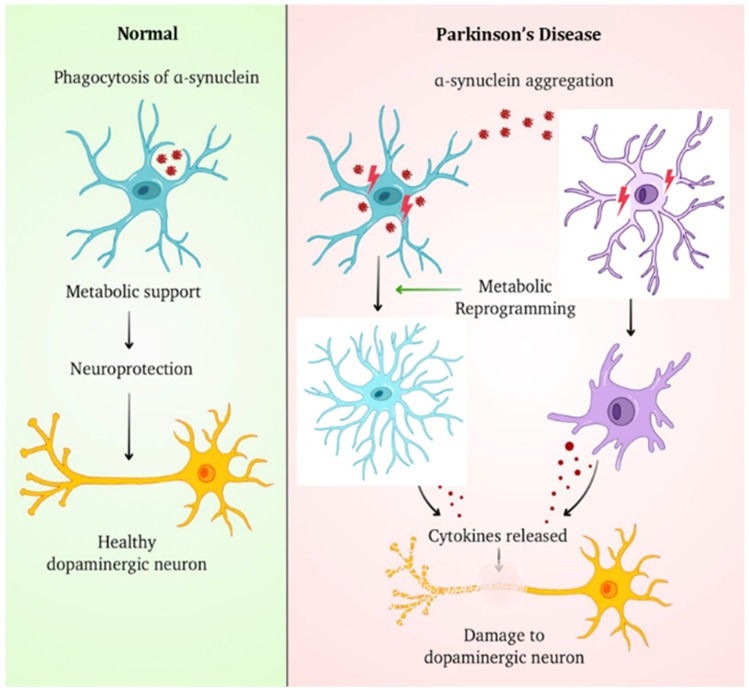
The role of glial cells in dopaminergic neuron health in both normal and Parkinson’s disease (PDD) conditions. The left panel shows how glial cells (blue) phagocytose α-synuclein, give metabolic support, and protect healthy dopaminergic neurons (yellow). Excessive α-synuclein aggregation in PD can cause glial cell dysfunction and metabolic reprogramming, as shown in the right panel. This activates astrocytes (light blue) and microglia (purple), causing the release of pro-inflammatory cytokines. These inflammatory mediators cause dopaminergic neuronal injury and degeneration, which is a characteristic of Parkinsonian neurodegeneration.

The metabolic reprogramming of glial cells also presents a novel target for therapeutic intervention. Research has indicated that glial cells can undergo metabolic shifts in response to neurotoxicants, which may impair their ability to support neuron health [110]. By restoring normal metabolic functions in glia, it may be possible to enhance their neuroprotective capabilities and improve outcomes in PD. This approach aligns with growing recognition of metabolic interplay between neurons and glial cells, where glial cells not only provide support but also actively participate in the metabolic demands of neurons [111]. Additionally, the involvement of enteric glial cells in PD is an emerging area of research. Given that altered gastrointestinal function is a common non-motor symptom observed in PD, understanding how enteric glial cells contribute to Gut–Brain signaling and neuroinflammation may provide insights into the disease’s pathogenesis [112]. The activation of enteric glial cells has been linked to neurodegenerative processes, suggesting that targeting these cells could offer a novel therapeutic avenue for treating both motor and non-motor symptoms of PD [113] (Figure 2).

In PD, α-synuclein aggregates engage with microglial TLR2/TLR4, initiating NF-κB-mediated cytokine secretion and the overexpression of iNOS and COX-2, both of which are associated with oxidative stress and dopaminergic neurotoxicity [34,114]. The chemokine (C-X3-C motif) ligand 1 (CX3CR1)–fractalkine signaling pathway, crucial for neuron–microglia communication, is compromised in PD models, resulting in microglial overactivation and an exacerbated M1 pro-inflammatory phenotype. Mice with MPTP lesions and CX3CR1 impairment demonstrate increased loss of dopaminergic neurons [115]. Astrocytes in PD exhibit compromised glutamate reuptake attributable to malfunctioning EAAT2 and the release of S100β, a neurotoxic protein that provokes oxidative stress. Activation of astrocytic JAK/STAT3 also contributes to neuroinflammation and astrogliosis [35].

While not pivotal to PD pathophysiology, oligodendrocyte dysfunction has been indicated by transcriptome analyses revealing diminished expression of myelin genes (MBP, MOG, PLP1) in the early stages of PD, potentially leading to white matter degeneration and synaptic instability [64]. Post-mortem analyses and in vivo imaging of PET tracers that target the 18 kDa TSPO, such as [^11^C]PK11195 and [^18^F] DPA-714, have consistently revealed elevated TSPO expression in the substantia nigra and striatum of individuals with PD. This rise indicates ongoing microglial activation, correlated with disease severity, particularly in the early and mid-stages of PD. These findings confirm microglial involvement not only as a subsequent response to neuronal death but also as a potential accelerator of dopaminergic degeneration [68].

In vivo, experimental models have provided further mechanistic insights. The MPTP-lesioned murine model, a frequently employed toxin-induced PD model, demonstrates rapid and selective degeneration of dopaminergic neurons. This is succeeded by notable microglial activation and the secretion of cytokines, including IL-1β, TNF-α, and interferon-γ. Pharmacological or genetic inhibition of TLR4, a microglial pattern recognition receptor, markedly diminishes neuroinflammation and neuronal apoptosis in these subjects, underscoring the contributory role of glia in neurodegeneration [59].

Recent findings suggest that astrocyte dysfunction contributes to the progression of PD by impairing glutamate uptake, calcium homeostasis, and the regulation of Blood–Brain Barrier permeability. Studies demonstrate reduced EAAT2 expression in astrocytes associated with PD, leading to excitotoxic neuronal damage. Moreover, α-synuclein generated by degenerating neurons is absorbed by astrocytes, initiating their reactive transformation and intensifying inflammation and oxidative stress [49].

In summary, glial cells especially microglia and astrocytes, are not merely reactive responders but are integral participants in the development of PD. Their role in inflammatory signaling, impaired protein clearance, excitotoxicity, and neurotrophic support makes them attractive targets for disease-modifying therapies. Enhanced understanding of peripheral glial networks, particularly intestinal glia, expands treatment options [116]. Future clinical strategies aimed at modifying glial phenotypes (transitioning from pro-inflammatory to neuroprotective states and enhancing glial-mediated trophic and metabolic support may yield more effective and personalized therapy for PD.

Furthermore, the mixed outcomes of glial-targeted strategies in PD exemplify the translational gap. The GDNF Phase II trial, while showing localized improvement in dopaminergic activity, failed to achieve its primary clinical endpoints, largely due to distribution limitations and patient heterogeneity [117]. These findings highlight how delivery across the BBB and sustained tissue penetration remain critical obstacles. Moreover, microglial activation in PD is highly context-dependent, shifting from potentially neuroprotective to overtly neurotoxic phenotypes as the disease progresses. Without reliable biomarkers to identify which patients might benefit from glial modulation, and at which stage, therapies risk being either ineffective or even detrimental. Addressing these challenges requires not only novel delivery technologies but also precision biomarker strategies that can guide intervention timing and patient selection. These findings mirror the shared translational constraints for glial-based therapies summarized in Section 9.1.

## 6. Huntington’s Disease (HD)

HD is an autosomal dominant, progressive neurodegenerative disorder presenting with motor dysfunction, cognitive decline, and psychiatric disturbances [70]. The mutation that occurs in the Huntingtin (*HTT*) gene results in an abnormal expansion of the CAG trinucleotide repeat sequence, encoding a polyglutamine stretch in the HTT protein. Individuals who have more than 35 CAG repeats in their *HTT* gene develop HD, with a longer repeat length correlating with early onset and severe disease progression [118]. The prevalence of HD is estimated at 10–13 cases per 100,000 individuals in populations with European ancestry and lower prevalence in Asian and African populations [119].

The clinical manifestation of HD is diverse, with the most recognizable feature being chorea, an involuntary, dance-like movement disorder. Other motor symptoms include dystonia, bradykinesia, and impaired gait and balance [118]. Cognitive impairment in HD progresses from subtle executive dysfunction in early stages to global dementia as the disease advances. Patients with HD also present psychiatric symptoms, including depression, anxiety, irritability, and apathy, which are common and often precede motor symptoms, which in turn significantly impact patients’ quality of life [120].

Pathologically, HD is marked by neuronal degeneration occurring in selective parts of the brain, particularly in the striatum, which includes the caudate nucleus and putamen, as well as in the cerebral cortex. This degeneration leads to disruptions in corticostriatal pathways, which are critical for motor, cognitive, and emotional functions [121]. The mutant HTT (mHTT) protein is prone to aggregation, forming intracellular inclusions that contribute to cellular toxicity and disrupt normal cellular functions, including transcriptional regulation, mitochondrial activity, and protein degradation pathways [122]. Currently, there is no cure for HD, and treatment is limited to symptomatic management. However, recent advances in molecular biology and genetics have paved the way for novel therapeutic strategies that target the various mechanisms involved in the disease process. These approaches include gene silencing therapies, such as antisense oligonucleotides and RNA interference, which aim to reduce the production of mHTT protein, and small-molecule drugs designed to enhance neuronal survival and function [123]. The ongoing research offers hope for disease-modifying treatments that could improve results for patients with HD.

### 6.1. Pathophysiology of HD

The abnormal CAG trinucleotide repeat expansion in the *HTT* gene leads to production of an abnormally long polyglutamine (polyQ) tract in the HTT protein, which undergoes abnormal post-translational modifications, misfolding, and proteolytic cleavage, generating toxic fragments that aggregate within neurons. These aggregates disrupt cellular functions such as transcriptional regulation, protein degradation, mitochondrial activity, and intracellular transport, contributing to neuronal death [122]. The dysfunction caused by mHTT is exacerbated by its abnormal interactions with various cellular proteins. Transcriptional dysregulation is an important key mechanism underlying the pathophysiology of HD. This mHTT binds to transcription factors, such as CREB-binding protein (CBP), disrupting their activity, leading to the downregulation of genes that are critical for neuronal survival, including those encoding BDNF, further increasing neuronal vulnerability [124]. Additionally, the disease impairs protein homeostasis mechanisms, such as the ubiquitin-proteasome system (UPS) and autophagy. These impairments result in the accumulation of misfolded and aggregated proteins, which create a feedback loop of cellular stress and dysfunction [60].

Mitochondrial dysfunction and oxidative stress are also hallmarks of HD pathophysiology. Mutation of *HTT* disrupts mitochondrial energy production and calcium buffering, leading to increased ROS and reduced ATP levels. These mitochondrial impairments exacerbate oxidative damage and neuronal injury [45]. Furthermore, excitotoxicity, driven by dysregulated glutamate signaling, plays a crucial role. Mutant *HTT* impairs astrocytic uptake of glutamate by affecting EAATs. This leads to excessive extracellular glutamate levels, resulting in overactivation of NMDA receptors, calcium overload, and neuronal death, particularly in the striatum [50].

Moreover, selective neuronal vulnerability is a defining feature of HD, with the striatum, particularly its medium spiny neurons (MSNs), being most affected. These neurons’ reliance on cortical inputs and their specific glutamate receptor profiles make them highly susceptible to excitotoxic damage. As the disease progresses, cortical regions also begin to degenerate, contributing to the cognitive and psychiatric symptoms observed in HD [125]. Additionally, neuroinflammation further exacerbates the disease process, wherein the activated microglia release pro-inflammatory cytokines and ROS, amplifying neuronal injury, while the astrocytic dysfunction also contributes to the inflammatory milieu and impairs neuronal support mechanisms [36]. The pathophysiology of HD involves a multifaceted interplay of toxic protein aggregation, disrupted cellular pathways, excitotoxicity, and neuroinflammation, culminating in progressive neuronal dysfunction and death. While the precise sequence of pathological events remains an area of active research, understanding these mechanisms provides insights into the development of potential therapeutic strategies that could slow or halt the disease progression.

### 6.2. The Role of Glial Cells in HD

Glial cells have a multifaceted role in the pathophysiology of HD and as a potential target for treating HD. Astrocytes are highly sensitive to mHTT, and studies have shown that mHTT disrupts astrocytic differentiation and function, leading to impaired neuroprotective capabilities and exacerbating neuronal damage. For instance, research indicates that astrocytic differentiation is hindered in glial progenitor cells expressing mHTT, which may delay synaptogenesis and circuit formation, contributing to the overall HD phenotype [37]. Furthermore, astrocytes have been shown to engage in neuroinflammatory processes that can further exacerbate neuronal dysfunction in HD [126]. The dysregulation of astrocytic functions, including cholesterol homeostasis and ion channel activity, has been linked to the pathogenesis of HD, suggesting that targeting these pathways could yield therapeutic benefits [51]. Microglia also play a vital role in HD as they are involved in the inflammatory response that characterizes the disease, and their prolonged activation leads to neuroinflammation, which is detrimental to the survival of neurons [37]. The interaction between microglia and astrocytes is critical, as both cell types can influence each other’s activation states and add to the overall inflammatory milieu in the brains of patients with HD [61]. Research has demonstrated that glial cells expressing mHTT can impart disease phenotypes to otherwise healthy neurons, indicating that glial pathology is a significant contributor to the neurodegenerative process in HD [127].

**Figure 3:**
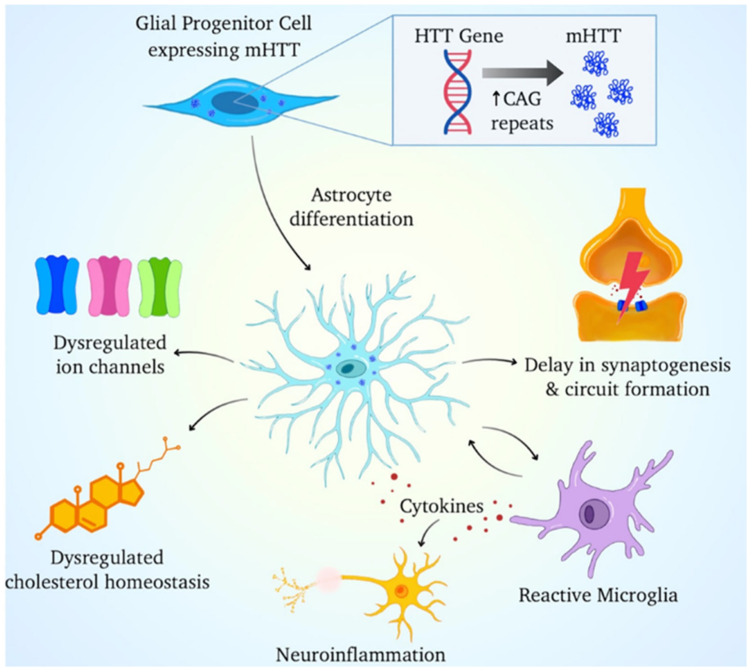
A Schematic illustration of how mutant huntingtin (mHTT) protein, resulting from increased CAG repeats in the HTT gene, affects glial progenitor cells and astrocyte differentiation. Astrocytes expressing mHTT exhibit multiple pathological features, including dysregulation of ion channels and cholesterol homeostasis, impaired synaptogenesis and neuronal circuit formation, and heightened cytokine release. These cytokines contribute to microglial activation and perpetuate neuroinflammation, ultimately leading to neuronal dysfunction and degeneration.

Therapeutically, glial cells present a new and promising avenue for intervention. For example, the infusion of glial conditioned medium has been shown to diminish the severity of pathology in mouse models of HD, suggesting that factors secreted by glial cells can exert protective effects on neurons [128]. Moreover, advancements in stem cell technology have opened possibilities for glial cell-based therapies, which could potentially restore normal glial function and mitigate the effects of mHTT on neuronal health [129]. The manipulation of gliogenic pathways and the restoration of normal glial function may provide novel strategies for improving outcomes in HD patients [65,71] (Figure 3). Collectively, these data indicate that malfunction in both astrocytes and microglia through disrupted ion and neurotransmitter balance and persistent cytokine signaling substantially contributes to the pathogenesis of HD and presents potential treatment options.

Although glial dysfunction is increasingly recognized as a key contributor to HD pathology, therapeutic attempts remain limited and face substantial barriers. Preclinical studies suggest that restoring astrocytic glutamate transport or modulating microglial reactivity can ameliorate neuronal dysfunction, yet translating these findings is complicated by interspecies differences in glial phenotypes and the difficulty of modeling progressive human disease [61]. Furthermore, HD exemplifies the challenge of context-dependent glial behavior: the same astrocytes that provide metabolic and synaptic support early in disease can become pro-inflammatory and neurotoxic in later stages. Without stage-specific biomarkers to capture these transitions, glial-targeted interventions risk being applied too broadly, reducing efficacy. Thus, future HD strategies must integrate longitudinal monitoring of glial phenotypes and patient stratification tools to ensure that glia-directed therapies are applied with maximal precision. The therapeutic implications align with the overarching barriers described in Section 9.1.

## 7. Multiple Sclerosis (MS)

MS is an immune-mediated, chronic, neurodegenerative disorder of the CNS in which the pathological features are neuroinflammation, demyelination, and neurodegeneration. It is the most common disabling neurological disease observed among young adults, typically occurring between the ages of 20 and 40, with a higher prevalence in women than in men [130]. There is geographical variation in the global prevalence of MS with higher prevalence rates in regions that are farther from the equator, likely reflecting a combination of genetic, environmental, and lifestyle factors [131]. The pathology of MS involves an aberrant immune response that targets the myelin sheath, which is the protective sheath surrounding axons in the peripheral nerves, as well as the oligodendrocytes responsible for myelin production in the CNS. This immune attack leads to the formation of demyelinated plaques in the CNS, primarily in white matter but also involving gray matter [132]. These plagues disrupt the normal conduction of electrical impulses along the affected nerves, resulting in the clinical symptoms of MS. Common manifestations include fatigue, motor and sensory deficits, visual disturbances, coordination and balance problems, and cognitive impairment [133]. The course of the disease is highly variable, ranging from relapsing-remitting episodes to a progressive form marked by gradual neurological decline.

Though the exact cause of MS remains unclear, it is believed to result from a complex relationship between genetic predisposition and environmental triggers. Over 200 genetic loci, including variations in the *HLA-DRB1* gene, have been shown to be associated with an increased risk of developing MS [134]. Environmental factors, such as latitude-dependent UV radiation exposure, Epstein–Barr virus infection, and smoking, have also been implicated in MS susceptibility [135]. Therapeutic advances in recent years have revolutionized the management of MS, particularly in relapsing-remitting forms of the disease. Various disease-modifying therapies (DMTs), such as interferon-beta, glatiramer acetate, and monoclonal antibodies like natalizumab and ocrelizumab, have been used with the aim to lower the frequency of occurrence and severity of relapses, slow disease progression, and mitigate CNS inflammation [136]. Emerging research is also focusing on remyelination therapies, neuroprotection, and strategies to repair damaged neurons and restore function. Despite these advancements, unmet needs remain, particularly for progressive forms of MS, underscoring the need for continued research and innovation in this field.

### 7.1. Pathophysiology of MS

MS is an immune-mediated, inflammatory, demyelinating, and neurodegenerative disorder. The disease begins with immune dysregulation, where autoreactive CD4+ T cells are activated against CNS antigens, influenced by genetic factors like the HLA-DRB1*15:01 allele and environmental triggers such as Epstein–Barr virus (EBV) infection and low vitamin D levels [137]. These activated T cells tend to cross through the Blood–Brain Barrier, which is facilitated by cytokines like TNF-α, allowing immune cells, including T and B cells, to infiltrate the CNS and initiate inflammation [138]. Once within the CNS, autoreactive T cells and B cells attack myelin, leading to demyelination. CD8+ T cells directly damage oligodendrocytes and axons, while B cells produce antibodies that enhance tissue destruction. This inflammation disrupts nerve conduction, leading to neurological symptoms [52].

Chronic inflammation results in oxidative stress, mitochondrial dysfunction, and axonal injury, driving neurodegeneration. Remyelination, initially mediated by oligodendrocyte precursor cells (OPCs), often fails in chronic stages due to an unfavorable inflammatory environment and loss of OPCs, leading to persistent demyelinated plaques [46]. Glial cells play a key role in MS. Microglia contribute to both repair and damage by clearing debris but releasing pro-inflammatory factors. Astrocytes form glial scars that limit further damage but also block remyelination, while oligodendrocyte loss exacerbates disease progression [52]. MS pathology shows active lesions with inflammation, chronic active lesions with a microglial rim, inactive demyelinated plaques, and partially repaired shadow plaques. These lesion types reflect the dynamic nature of MS and its progression [138].

### 7.2. The Role of Glial Cells in MS

Glial cells play a multifaceted role in the pathophysiology of MS, contributing both to disease progression and to potential avenues for repair. The primary glial cell types involved, astrocytes, oligodendrocytes, and microglia, each exert unique influences on inflammation, demyelination, and remyelination. Astrocytes are pivotal regulators of neuroinflammation in MS, yet microglia remain the main contributors to the initiation and propagation of inflammatory cascades, as emphasized by recent evidence.”. They secrete cytokines and chemokines, such as CCL2 and CXCL10, which recruit immune cells across the BBB and sustain inflammatory responses [38]. Activated astrocytes also undergo reactive gliosis, producing glial scars that impede remyelination and axonal regeneration [139]. Key intracellular pathways, including JAK/STAT3 and NF-κB, are upregulated in astrocytes within MS lesions, amplifying inflammatory signaling and fostering chronic lesion development [140,141]. Because of their central role in modulating both BBB integrity and immune infiltration, astrocytes represent a compelling therapeutic target, where modulation of their inflammatory cascades may mitigate CNS damage.

Oligodendrocytes, the myelinating cells of the CNS, are directly targeted in MS, and their loss severely compromises neuronal function [142]. Although OPCs are abundant in MS lesions, they exhibit impaired differentiation in progressive forms of the disease. This blockade is partly driven by aberrant activation of developmental pathways such as Wnt and Notch, which maintain OPCs in an undifferentiated state [53]. Additionally, immune-derived factors, including TNF-α and FasL, induce oligodendrocyte apoptosis, further undermining remyelination [66]. Enhancing OPC maturation and protecting oligodendrocytes from immune-mediated injury remain central goals for restorative therapies. Microglia also play a dual role in MS, oscillating between pro-inflammatory and reparative phenotypes depending on lesion stage and microenvironmental cues [62]. Pro-inflammatory microglia (M1-like) release IL-1β, TNF-α, and reactive oxygen species, driving axonal injury and oligodendrocyte death [39]. Moreover, activation of the NLRP3 inflammasome exacerbates CNS inflammation and demyelination, both in human lesions and experimental autoimmune encephalomyelitis (EAE) models [40]. Conversely, anti-inflammatory microglia can promote debris clearance and remyelination, suggesting that strategies aimed at skewing microglia toward reparative states may hold therapeutic promise. Importantly, cross-talk between microglia and astrocytes further shapes the inflammatory milieu, amplifying or attenuating neuroinflammation depending on context [143].

**Figure 4:**
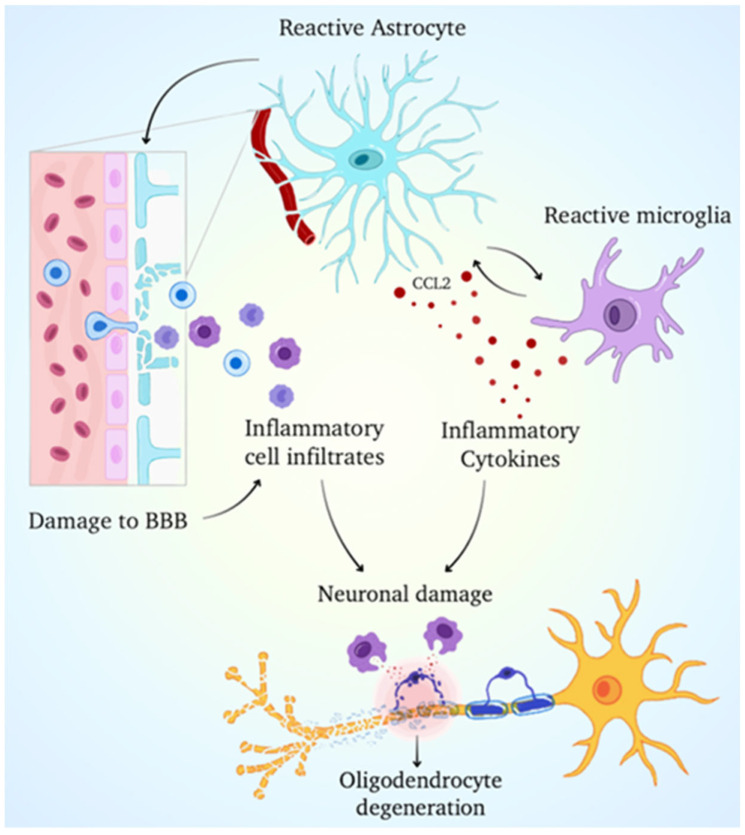
Schematic illustration represents the neuroinflammatory events that occur as a result of Blood–Brain Barrier disruption. The breakdown of the Blood–Brain Barrier allows peripheral inflammatory cells to enter the central nervous system. This activates astrocytes and microglia, resulting in the release of pro-inflammatory cytokines such as CCL2. These cytokines promote microglial activation and exacerbate the inflammatory response. The persistent inflammatory environment causes neuronal damage and contributes to neurodegeneration. This graphic depicts the relationship between glial activation, BBB integrity, and inflammatory signaling in central nervous system pathology.

Recent advances in stem cell and glial biology offer additional opportunities for therapy. Induced pluripotent stem cell (iPSC)-derived glial models provide patient-specific platforms to study disease mechanisms and test interventions [72]. Emerging approaches such as cell-penetrating peptides capable of targeting astrocytes and oligodendrocytes may improve drug delivery directly to affected glial populations (Figure 4). Collectively, these innovations highlight the therapeutic relevance of glia in MS, while also underscoring the complexity of targeting cells with context-dependent and dynamic functions.

Yet, the therapeutic promise of glial modulation in MS is tempered by several translational challenges. While astrocytic modulation and remyelination strategies show efficacy in animal models, the dynamic and stage-specific nature of glial phenotypes in humans complicates their clinical application [144]. Reactive astrocytes, for instance, may support tissue repair in some contexts but generate inhibitory scars in others, while microglial responses vary dramatically depending on lesion stage [145]. Moreover, the lack of precise biomarkers to distinguish beneficial from detrimental glial states hampers patient stratification and trial design [146]. These obstacles parallel inconclusive outcomes in other neurodegenerative diseases, such as AD and PD, and highlight the need for advanced imaging, molecular fingerprinting, and patient-derived models to guide therapy. Without such precision tools, the translation of promising glial-targeted strategies for MS risks being constrained by the same pitfalls that have limited progress in other conditions. These challenges correspond to the common translational limitations detailed in Section 9.1.

## 8. Amyotrophic Lateral Sclerosis (ALS)

ALS, known as Lou Gehrig’s disease, is a neurodegenerative disorder that is progressive in nature and affects the motor neurons of the brain and spinal cord. The disease presents muscle weakness, atrophy, and eventual paralysis. The disease primarily affects the upper motor neurons (UMNs) in the motor cortex of the brain and lower motor neurons (LMNs) of the brainstem and spinal cord. The result is the progressive loss of voluntary muscle control, which leads to fatal respiratory failure [47]. The global incidence of ALS is approximately 1.75 per 100,000 person-years and shows a slightly higher prevalence among men than women [147]. Most cases are sporadic, accounting for 90–95% of cases, while 5–10% are familial, associated with genetic mutations. Common genetic causes include mutations in the SOD1, C9ORF72, TARDBP, and FUS genes. Environmental factors, such as exposure to toxins, traumatic head injuries, and military service, have been indicated as potential risk factors [148].

Pathological features of ALS include loss of motor neurons, gliosis, and accumulation of misfolded proteins, including TAR DNA binding protein 43 (TDP-43), and SOD1 accumulation in motor neurons. These pathological changes result in the degeneration of motor pathways, leading to the clinical manifestations of ALS, such as spasticity, muscle weakness, and hyperreflexia in UMN involvement, and fasciculations, muscle atrophy, and hyporeflexia in LMN involvement [41]. Despite significant advances in understanding ALS pathophysiology, its exact cause remains unclear, and the disease lacks a definitive cure. Current therapeutic approaches, including riluzole and edaravone, provide modest benefits in slowing disease progression. Ongoing research focuses on exploring novel therapeutic targets, such as glial cell modulation and gene therapies, to address the unmet clinical need [47].

### 8.1. Pathophysiology of ALS

ALS pathology exhibits neurodegeneration with progressive loss of motor neurons in the brain, brainstem, and spinal cord. Its pathophysiology involves a combination of various genetic, molecular, and environmental factors which produce motor neuron dysfunction and degeneration. Among these, C9ORF72 repeat expansions and SOD1 mutations are the most studied, with the former causing RNA toxicity and dipeptide repeat aggregation, and the latter contributing to oxidative stress through protein misfolding. Sporadic ALS (sALS) is believed to be caused by a combination of genetic predisposition and environmental factors, such as exposure to toxins, oxidative stress, and excitotoxicity [41,148].

Protein misfolding and aggregation are key pathological features of ALS. Aggregates of TDP-43 have been observed in the cytoplasm of affected neurons, disrupting RNA processing, protein homeostasis, and axonal transport. Misfolded SOD1 protein further exacerbates oxidative damage and mitochondrial dysfunction. These changes impair energy production and trigger apoptosis, leading to motor neuron degeneration [47]. Excitotoxicity also plays a critical role in ALS pathophysiology. Impaired glutamate clearance by astrocytes, due to reduced activity of the EAAT2), leads to excessive glutamate signaling, causing calcium influx, mitochondrial overload, and neuronal injury [54].

Neuroinflammation is another significant contributor to ALS progression. Microglia and astrocytes, which are activated during the disease process, release pro-inflammatory cytokines and ROS, which in turn exacerbate neuronal damage. While glial cells have an essential part in the process of clearing debris and maintaining homeostasis, their chronic activation leads to neurotoxicity and a failure in providing adequate trophic support for the motor neurons [42]. Additionally, defects in axonal transportation mechanisms impair the delivery of essential organelles and molecules along the axons of motor neurons, disrupting synaptic function and retrograde signaling. These disruptions contribute to the characteristic distal-to-proximal pattern of neurodegeneration observed in ALS, where neuromuscular junctions are initially denervated, leading to muscle atrophy and further motor neuron death [149].

Overall, the pathophysiology of ALS is multifactorial, involving genetic mutations, protein misfolding, oxidative stress, excitotoxicity, neuroinflammation, and axonal transport defects. These mechanisms interact dynamically to drive progressive motor neuron degeneration and the associated clinical manifestations of ALS.

### 8.2. Role of Glial Cells in ALS

Glial cells make a significant contribution to the pathophysiology of ALS and represent a potential target for developing new treatments. Again, the multifaceted roles of glial cells, in which the astrocytes and microglia have been implicated in both the progression of the ALS disease process and their importance in the development of therapeutic strategies. The neuroinflammatory response has been recognized as a significant pathological feature of ALS, suggesting that glial cells are not merely passive participants but active contributors to the disease process [150,151].

**Figure 5:**
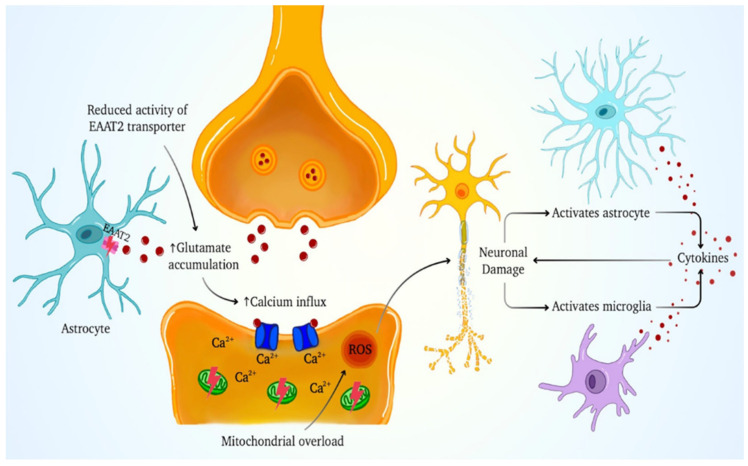
The schematic illustration depicts the neurotoxic processes that begin with diminished activity of the EAAT2 (excitatory amino acid transporter 2) on astrocytes, resulting in impaired glutamate uptake. Excess extracellular glutamate stimulates postsynaptic glutamate receptors and increases calcium input into neurons, causing mitochondrial calcium overload and increased generation of reactive oxygen species (ROS). This causes neuronal injury, which then activates astrocytes and microglia. The activated glial cells release pro-inflammatory cytokines, exacerbating neuroinflammation and neuronal damage.

Studies using mutant SOD1 mice, a common model for ALS, have demonstrated that glial cells can exhibit toxic effects on motor neurons, indicating that targeting glial activation may provide therapeutic benefits [63]. Research has shown that modulating specific receptors on glial cells, such as the prostaglandin D2 receptor subtype DP1, can lead to prolonged survival in ALS models [152]. This suggests that interventions aimed at reducing the inflammatory response or altering glial cell function could ameliorate disease progression. Additionally, the transplantation of glial-restricted progenitors has shown promise in preserving neuronal function and promoting recovery in models of spinal cord injury, which may have implications for ALS treatment strategies [67]. The role of glial cells in neuroprotection is gaining attention. Activated astrocytes can produce neurotrophic factors that support neuronal survival and cellular repair mechanisms [73]. This neuroprotective role is critical, as enhancing the supportive functions of astrocytes could counteract the degenerative processes occurring in ALS. The interplay between glial cells and neurons is complex, with glial cells also involved in synaptic regulation and homeostasis, which are vital for neuronal health [74] (Figure 5).

Furthermore, in ALS, glial cells clearly contribute to motor neuron degeneration, yet therapeutic translation remains elusive. Attempts to modulate astrocytic glutamate transport (e.g., with ceftriaxone to enhance EAAT2 expression) have shown benefit in animal models but failed to produce consistent clinical results, reflecting the difficulty of sustaining glial modulation in humans [11]. Similarly, microglial-targeted interventions face the challenge of balancing protective debris clearance with detrimental pro-inflammatory activity, which shifts dynamically over the disease course. The absence of biomarkers capable of distinguishing these glial states hampers trial design and dilutes efficacy signals. Moreover, the safety of long-term glial modulation raises concerns, as prolonged suppression of microglial activity may impair immune surveillance. These limitations underscore the urgent need for biomarker-driven patient stratification and precision timing of glial interventions if ALS therapies are to succeed in translation. Similar cross-cutting translational issues are outlined in Section 9.1

## 9. Glial-Targeted Therapeutics and Clinical Trials

### 9.1. Challenges and Limitations of Glial-Targeted Therapies

Despite encouraging outcomes in preclinical studies, glial-targeted therapies face persistent translational obstacles that have limited their clinical success. A central challenge lies in the dynamic and context-dependent nature of glial phenotypes. Microglia, for instance, can adopt protective states that promote debris clearance and trophic support in early diseases, but shift toward pro-inflammatory, neurotoxic profiles as pathology advances [68]. Similarly, astrocytes oscillate between neuroprotective (A2) and neurotoxic (A1) states depending on local signals [21]. These shifting roles complicate therapeutic targeting and highlight the need for interventions that are tailored to disease stage and glial subtype rather than applied universally. In addition, preclinical models often fail to recapitulate human glial biology. Rodents differ significantly from humans in gene expression and glial reactivity, which diminishes the predictive value of animal studies and contributes to the translational gap. Emerging techniques, including astrocyte-to-neuron reprogramming, have shown functional neuronal recovery in rodent models of stroke and AD by the application of transcription factors (e.g., NeuroD1, Sox2, Ascl1). Furthermore, interspecies variations in glial gene expression and reactivity diminish the reliability of mouse models in representing human illness. Unsuccessful or inconclusive trials—exemplified by the Phase IIb GDNF trial in PD, which demonstrated no meaningful clinical enhancement in the primary outcomeunderscore this translational gap and emphasize the necessity for more predictive platforms [117].

Another critical limitation is the absence of reliable biomarkers to stratify patients and monitor therapeutic responses. Despite advancements in PET tracers for TSPO and other microglial markers, identifying glial activation states or subtypes in vivo continues to be problematic. In the absence of precise biomarkers, trials may include disparate patient populations, hence diminishing efficacy signals. Extended inhibition of microglial activation may hinder immune surveillance and tissue healing, whereas indiscriminate manipulation of astrocytes could disturb neurotransmitter cycling and ionic equilibrium.

Finally, challenges in drug delivery and safety remain significant barriers. The BBBrestricts the entry of many candidate compounds, while the diffuse distribution of glia across the CNS makes sustained and selective modulation difficult. Moreover, long-term interventions carry risks: prolonged inhibition of microglia may compromise immune surveillance, and indiscriminate manipulation of astrocytes could disrupt neurotransmitter cycling and ionic balance.

Taken together, these limitations underscore the need for a precision medicine approach to glial therapeutics. Future strategies must integrate biomarker-guided patient selection, stage-specific interventions, and improved humanized models to enhance translatability. By addressing these challenges directly, the field can move beyond repeated failures and toward the development of safe, effective, and truly disease-modifying therapies.

### 9.2. Therapeutics Targeting Glial Cells and Clinical Trials

Recently, the involvement of glial cells in neurodegeneration has prompted an increase in treatment strategies designed to modify glial activities. These interventions encompass monoclonal antibodies, small compounds, gene therapies, and reprogramming technologies, many of which are at different phases of clinical or preclinical research.

TREM2 has become a significant therapeutic target in AD. TREM2 activation improves microglial survival, chemotaxis, and Aβ phagocytosis. Agonistic anti-TREM2 antibodies, including AL002 (Alector/AbbVie) and ABBV-CLS-7262, are presently undergoing Phase 2 clinical trials (INVOKE-2 and INFRONT-3) to redirect microglial activity towards a disease-associated protective phenotype while mitigating pro-inflammatory damage [30].

Another target is CSF1R (Colony-Stimulating Factor 1 Receptor), which governs microglial growth and activation. CSF1R inhibitors such as PLX3397 (pexidartinib) and JNJ-40346527 have shown efficacy in depleting microglia and diminishing neuroinflammation in models of AD and ALS; however, the long-term implications for immune surveillance are still ambiguous [153].

Astrocytes are now acknowledged for their essential functions in glutamate clearance, synaptic support, and the maintenance of the BBB. Attempts to enhance astrocytic glutamate transport by the overexpression of EAAT2 via ceftriaxone have demonstrated neuroprotective effects in ALS animal models, while clinical results are yet constrained [55].

The reprogramming of reactive astrocytes into functioning neurons has been successful in mouse models using the viral delivery of transcription factors, including NeuroD1, Sox2, or Ascl1. These methodologies have shown the ability to reverse behavioral and cognitive impairments in models of AD and stroke [154]. Nonetheless, the clinical application is limited by apprehensions over safety, delivery efficacy, and long-term stability [105].

## 10. Conclusions

Glial cells—astrocytes, microglia, and oligodendrocytes are central drivers of neurodegeneration, shaping inflammation, synaptic health, and neuronal survival across AD, PD, HD, MS, and multiple system atrophy. Although mechanistic insights have revealed compelling targets such as TREM2, EAAT2, and Wnt/Notch signaling, clinical translation has been disappointing. The failures of anti-TREM2 antibodies, GDNF delivery, and other glial-targeted strategies illustrate recurring barriers: the dynamic and stage-dependent nature of glial phenotypes, limited biomarker tools for patient stratification, poor cross-species translatability, and challenges in CNS drug delivery. These setbacks highlight not a lack of therapeutic potential, but the urgent need for precision glial medicine. Future progress depends on biomarker-guided approaches to distinguish protective from detrimental glial states, patient-derived models to improve translational fidelity, and stage-specific interventions that reflect the shifting roles of glia over the disease course. Emerging innovations from astrocyte reprogramming to microglial repopulation illustrate the path forward. By learning from past failures and refining strategies accordingly, the field can transform glial modulation from a promising concept into truly disease-modifying therapies for currently intractable neurodegenerative disorders.

## Figures and Tables

**Table 1:** Common glial-mediated mechanisms in major neurodegenerative diseases.

Glial Mechanism	AD	PD	HD	MS	ALS
**Chronic neuroinflammation**	Microglial and astrocytic activation via Aβ and tau, NLRP3 inflammasome, NF-κB pathway [31,32]	Microglial activation by α-synuclein via TLR2/TLR4; astrocytic JAK/STAT3 signaling [33,34,35]	Pro-inflammatory microglial response to mHTT; NF-κB activation [36,37]	Microglial M1 polarization, inflammasome activation, astrocytic cytokine release [38,39,40]	SOD1/TDP-43 pathology triggers glial cytokine release; toxic gain-of-function in microglia [41,42]
**Oxidative stress**	ROS from reactive astrocytes and microglia; mitochondrial dysfunction [43]	Microglial-derived ROS and iNOS expression; astrocytic glutamate toxicity [34,44]	Increased ROS from dysfunctional mitochondria and glia [36,45]	ROS from M1 microglia and reactive astrocytes [39,46]	Glial-derived ROS; impaired antioxidant defense [41,47]
**Glutamate dysregulation/excitotoxicity**	Astrocytic EAAT2 downregulation → synaptic toxicity [9]	EAAT2 dysfunction; excess glutamate → dopaminergic toxicity [48,49]	Reduced EAAT2 and Kir4.1 in astrocytes → striatal excitotoxicity [50,51]	Altered glutamate transport contributes to demyelination [52,53]	EAAT2 loss in astrocytes → MN excitotoxicity [54,55]
**Impaired protein clearance**	Microglial failure to clear Aβ; impaired autophagy [9,56]	Astrocytic/microglial clearance of α-synuclein aggregates [57,58,59]	Deficient glial clearance of mHTT; impaired UPS/autophagy [37,60,61]	Impaired debris clearance delays remyelination [52,62]	Failure to clear misfolded SOD1, TDP-43; autophagy dysregulation [11,41,63]
**Myelin and metabolic dysfunction**	Astrocyte and oligodendrocyte dysfunction impair white matter integrity [20]	Early oligodendrocyte gene dysregulation, reduced myelin proteins [64]	Altered cholesterol and metabolic support by astrocytes [51,65]	Direct oligodendrocyte loss; impaired OPC differentiation [53,66]	Axonal demyelination; oligodendrocyte metabolic stress [47,67]
**Aging-related glial changes**	Increased inflammatory bias; decreased phagocytosis [68]	Age-primed microglia → heightened sensitivity to insults [69,70]	Glial senescence contributes to disease progression [71]	Age reduces the remyelination capacity of OPCs [72]	Aging enhances glial activation and reduces neurotrophic support [73,74]

Aβ, amyloid beta; AD, Alzheimer’s Disease; PD, Parkinson’s Disease; HD, Huntington’s Disease; MS, Multiple Sclerosis; ALS, Amyotrophic Lateral Sclerosis. (NLRP3: Nucleotide-Binding Domain, Leucine-Rich Repeat-Containing Protein 3, NF-κB: Nuclear factor kappa-light-chain-enhancer of activated B cells, ROS: Reactive oxygen species, EAAT2: Excitatory Amino Acid Transporter 2, TLR2/TLR4: Toll-like receptor 2 (TLR2) and Toll-like receptor 4, JAK/STAT3: Janus kinase (JAK)/signal transducer and activator of transcription 3, iNOS: Inducible Nitric Oxide Synthase, mHTT: mutant huntingtin protein, SOD1: Superoxide Dismutase 1, TDP-43: TAR DNA-Binding Protein 43).

## Data Availability

No new data were created or analyzed in this study.

## Abbreviations

The following abbreviations are used in this manuscript:

AD	Alzheimer’s disease
AKT	Protein kinase B
ALS	Amyotrophic lateral sclerosis
ApoE	Apolipoprotein E
APP	amyloid precursor protein
ATP	Adenosine triphosphate
Aβ	amyloid-beta plaques
BBB	Blood–brain barrier
BDNF	Brain-derived neurotrophic factor
C9ORF72	Chromosome 9 open reading frame 72
AA	Cerebral amyloid angiopathy
CAG	Cytosine, Adenine, Guanine trinucleotide
CBP	CREB-binding protein
CCL2	Chemokine (C-C motif) ligand 2
CD4 cells	Clusters of differentiation 4
CD8	Clusters of differentiation 8
CNS	Central nervous system
*CREB*	cAMP-response element binding *protein*
DMTs	Disease-modifying therapies
DP1	D-type prostanoid receptor 1
EAATs	Excitatory amino acid transporters
EBV	Epstein–Barr virus
FALS	Familial Amyotrophic Lateral Sclerosis
FUS	Fused in sarcoma genes
GDNF	Glial cell line-derived neurotrophic factor
GFAP	Glial fibrillary acidic protein
HLA-DRB1	Human Leucocyte Antigen DR Beta 1
HTT	Huntingtin gene
iPSCs	Induced pluripotent stem cells
IR4.1	Inwardly rectifying potassium channel 4.1
LMNs	Lower motor neurons
LRRK2	Leucine-rich repeat kinase 2
LTD	long-term depression
LTP	long-term potentiation
mHTT	Mutant huntingtin protein
MS	Multiple sclerosis
MSNs	Medium spiny neurons
NF-κB	Nuclear factor kappa B
NFTs	neurofibrillary tangles
NMDA	N-methyl-D-aspartate
OPCs	Oligodendrocyte precursor cells
PARK7	Parkinsonism Associated Deglycase
PD	Parkinsons disease
PI3K	Phosphatidylinositol 3-kinase
PolyQ	Polyglutamine
PSEN1	Presenilin-1
PSEN2	Presenilin-2
RNA	Ribonucleic acid
ROS	Reactive oxygen species
sALS	Sporadic ALS
SNCA	Synuclein alpha
sNpc	Substantia nigra pars compacta
SOD1	Superoxide dismutase type 1
TARDBP	Transactive response DNA-binding protein
TDP-43	Transactive response DNA-binding protein of 43 kDa
TNF-α	Tumor necrosis factor alpha
UMNs	Upper motor neurons
PS	Ubiquitin-proteasome system
UV	Ultraviolet r
